# Evidence of Zika Virus Reinfection by Genome Diversity and Antibody Response Analysis, Brazil

**DOI:** 10.3201/eid3002.230122

**Published:** 2024-02

**Authors:** Marcia da Costa Castilho, Ana Maria Bispo de Filippis, Lais Ceschini Machado, Thaise Yasmine Vasconcelos de Lima Calvanti, Morganna Costa Lima, Vagner Fonseca, Marta Giovanetti, Cassia Docena, Armando Menezes Neto, Camila Helena Aguiar Bôtto-Menezes, Edna Oliveira Kara, Rafael de La Barrera, Kayvon Modjarrad, Silvana Pereira Giozza, Gerson Fernando Pereira, Luiz Carlos Junior Alcantara, Nathalie Jeanne Nicole Broutet, Guilherme Amaral Calvet, Gabriel Luz Wallau, Rafael Freitas Oliveira Franca

**Affiliations:** Tropical Medicine Foundation Doctor Heitor Vieira Dourado, Manaus, Brazil (M. da Costa Castilho, C.H.A. Bôtto-Menezes);; Oswaldo Cruz Foundation, Rio de Janeiro, Brazil (A.M.B. de Filippis, M. Giovanetti, L.C.J. Alcantara);; Oswaldo Cruz Foundation, Recife, Brazil (L.C. Machado, T.Y.V. de L. Calvanti, M.C. Lima, C. Docena, A.M. Neto, G.A. Calvet, G.L. Wallau, R.F.O. Franca);; Organização Pan-Americana da Saúde/Organização Mundial da Saúde, Brasília, Brazil (V. Fonseca);; University of Campus Bio-Medico di Roma, Rome, Italy (M. Giovanetti);; World Health Organization, Geneva, Switzerland (E.O. Kara, N.J.N. Broutet);; Walter Reed Army Institute of Research, Silver Spring, Maryland, USA (R. de La Barrera, K. Modjarrad);; Department of Chronic Condition Diseases and Sexually Transmitted Infections, Brasília, Brazil (S.P. Giozza, G.F. Pereira);; National Reference Center for Tropical Infectious Diseases, Hamburg, Germany (G.L. Wallau)

**Keywords:** Zika virus, viruses, vector-borne infections, genetic diversity, evolution, epidemiology, reinfection, Brazil

## Abstract

We generated 238 Zika virus (ZIKV) genomes from 135 persons in Brazil who had samples collected over 1 year to evaluate virus persistence. Phylogenetic inference clustered the genomes together with previously reported ZIKV strains from northern Brazil, showing that ZIKV has been remained relatively stable over time. Temporal phylogenetic analysis revealed limited within-host diversity among most ZIKV-persistent infected associated samples. However, we detected unusual virus temporal diversity from >*5* persons, uncovering the existence of divergent genomes within the same patient. All those patients showed an increase in neutralizing antibody levels, followed by a decline at the convalescent phase of ZIKV infection. Of interest, in 3 of those patients, titers of neutralizing antibodies increased again after 6 months of ZIKV infection, concomitantly with real-time reverse transcription PCR re-positivity, supporting ZIKV reinfection events. Altogether, our findings provide evidence for the existence of ZIKV reinfection events.

Zika virus (ZIKV) is an arthropodborne virus belonging to the Flaviviridae family. Virions are enveloped for a single-stranded positive-sense RNA genome of ≈10.8-kb (*1*). ZIKV is transmitted through the bite of infected *Aedes* spp. mosquitoes, mainly *A. aegypti*, which are widely distributed throughout the tropical and subtropical regions of the world.

In 2015, a large ZIKV epidemic was documented in Brazil, resulting in an estimated 440,000–1.3 million cases (*2*). Of great concern, the epidemic was preceded by a dramatic increase in the number of congenital anomalies, including newborn microcephaly (*3*,*4*). However, since the largest outbreak in 2015, ZIKV has decreased its circulation; novel cases are only sporadically reported (*5*).

ZIKV infections are usually asymptomatic, although a small proportion of persons may experience mild symptoms such as fever, rash, nonpurulent conjunctivitis, muscle pain, and joint pain. During pregnancy, ZIKV infection may result in microcephaly and other congenital abnormalities in the developing fetus (*3*). Suspected cases are diagnosed by detection of viral RNA in blood and urine during the acute phase of the disease and in other body fluids with variable frequency and duration by reverse transcription PCR (RT-PCR) (*6*). As previously reported, ZIKV infection may result in a persistent viral infection, as demonstrated by the prolonged detection of viral RNA in semen; the longest detection was up to 370 days after symptom onset (*7*). Virus compartmentalization and persistence are common features of ZIKV infection; however, the clinical and immunological aspects of ZIKV persistence, reactivation, and reinfection are still unknown.

ZIKV phylogenetic studies have described the circulation of 2 distinct African and Asian lineages (*8*). The initial genetic analysis of the first ZIKV isolates from Brazil revealed the circulation of the Asian genotype during the 2015–2016 epidemic (*9*). Asian-derived strains that currently circulate in the Americas are now named ZIKV American strains and are well known for their capacity to infect neuronal progenitor cells, disrupting cell development, proliferation, and differentiation (*10*,*11*). Because the genomic replication of ZIKV is based on an error-prone RNA-dependent RNA polymerase (RdRp), which leads to nucleotide misincorporation during viral replication, ZIKV infection behaves as viral populations composed of genetically related sequences, similar to other RNA virus infections. As the viral replication progresses in an infected person, mutations start to accumulate, resulting in more heterogeneous viral genomic populations. Those viral population clouds are the foundation of the quasispecies theory, which posits that RNA viruses produce larger, highly variable population clouds that can evade the host immune system more efficiently (*12*). Furthermore, accumulating data show that viral cloud variability is able to interfere with disease progression (*13*,*14*). In this context, next-generation sequencing (NGS) provides a powerful tool to gain a deeper understanding of viral diversity by increasing the depth of sequencing coverage, defined as the number of reads for a given nucleotide). Therefore, the assessment of viral diversity is key to better understand virulence, evolution, and host-specific adaptations providing a direct translational information to mitigate effects of viral pathogens.

In this study, we deployed an NGS protocol to gain insight into the genetic diversity of ZIKV in naturally infected patients. Specifically, we used a previously established cohort study conducted in northern Brazil to assess virus diversity from patients with prolonged ZIKV infection (*15*–*17*). Since 2016, we have observed limited virus diversity and decreasing ZIKV transmission over the years, which was likely because of population immunity elicited during the first outbreak waves. We also found that virus diversity was limited in longitudinally sequenced samples from persons persistently infected with ZIKV, indicating restrained evolutionary rates and selection pressures acting on RNA arthropod-borne viruses; our results were consistent with previously published findings (*18*,*19*). However, we also detected the existence of divergent genomes within the same patient in a small number of samples analyzed; those participants responded to infection with alterations in neutralizing antibodies levels concomitantly with ZIKV redetection by real-time RT-PCR (rRT-PCR) several months after the initial virus exposure.

The study protocol and procedures have been reviewed and approved by the World Health Organization Research Ethics Review Committee (protocol ID: ERC.0002786); Brazilian National Research Ethics Commission (CAAE: 62.518.016.6.1001.0008); Institutional Ethics and Research Committee of the Evandro Chagas National Institute of Infectious Diseases, Fiocruz, Rio de Janeiro (CAAE: 62.518.016.6.2002.5262); Institutional Ethics and Research Committee of the Aggeu Magalhães Research Center, Fiocruz, Recife (CAAE: 62.518.016.6.2001.5190) and Institutional Ethics and Research Committee of the Tropical Medicine Foundation, Manaus, Amazonas (CAAE: 62.518.016.6.2003.0005).

## Methods

### Study Participants and Specimen Collection

Participants comprised men and women >18 years of age with a confirmed diagnosis of ZIKV infection by RT-PCR, as described previously (*15*,*16*,*20*). Participants were persons with symptomatic cases diagnosed at the study collaborating clinics (index case-patients) and their asymptomatic or symptomatic household and sexual contacts. After ZIKV infection confirmation performed 48 hours after study recruitment, we collected other specimens at established intervals, or visits (Table 1), and routinely tested for molecular screening using a multiplex rRT-PCR assay to detect ZIKV, dengue virus, and chikungunya virus.

**Table 1:** Study visits and sample collection for participants in study of Zika virus reinfection, Brazil

Visit no.	Days after rash, range*
V0	–7 to 0
V1	0–2
V2	3–4
V3	5–8
V4	9–10
V5	11–20
V6	21–30
V7	31–60
V8	61–90
V9	91–120
V10	121–150
V11	151–180
V12	181–210
V13	211–240
V14	241–270
V15	271–300
V16	301–330
V17	331–360

*As reported by study participants.

### NGS and Analysis

We processed all specimens with a positive ZIKV rRT-PCR result, defined as a cycle threshold (Ct) value <38, using a previously established NGS protocol (*21*). For this study, we processed plasma, urine, and semen samples (semen is more frequently associated with persistence). For sequencing, we first obtained a complementary DNA employing the ProtoScript II Reverse transcription kit (New England Biolabs, https://www.neb.com) and a set of random primers (random sequence [d(N)6]). We obtained whole-genome amplicons from a multiplex PCR reaction using a set of ZIKV designed primers, as described by Quick et al. (*21*); we purified amplicons using the Q5 High-Fidelity DNA Polymerase kit (New England Biolabs) and performed library preparation with the Nextera XT Library Prep kit (Illumina, https://www.illumina.com) using 2 ng of DNA. We sequenced the obtained libraries using the MiSeq Reagent kit version 3 (Illumina) on an Illumina MiSeq. We processed raw fastq data to generate consensus files (base calls only at regions with >5×) and to call SNVs and iSNVs (only regions with a coverage depth of >100×) using ViralFlow version 0.0.6 (*22*) and a reference ZIKV genome (GenBank accession no. KX197192.1).

### Phylogenetic and Bayesian Analysis

The new genomic sequences reported in this study were initially submitted to a genotyping analysis using the ZIKV typing tool (http://genomedetective.com/app/typingtool/zika). We aligned genomic data generated in this study (238 genomes with coverage breadth >70 and average coverage depth of 100×) with a worldwide dataset of ZIKV genome sequences (n = 840 for all known ZIKV genotypes and n = 481 for ZIKV American strains). We aligned sequences using MAFFT (https://mafft.cbrc.jp/alignment/software) and inferred a preliminary maximum-likelihood tree using IQ-TREE version 2 (http://www.iqtree.org). Before conducting temporal analysis, we assessed our dataset for molecular clock signal in TempEst version 1.5.3 (http://tree.bio.ed.ac.uk/software/tempest) after removing any potential outliers that might violate the molecular clock assumption. To estimate a time-calibrated phylogeny, we used the Bayesian software package BEAST version 1.10.4 (https://beast.community) with the Bayesian skyline tree prior with an uncorrelated relaxed clock and the lognormal distribution. We ran analyses in duplicate in BEAST for 100 million Markov chain Monte Carlo (MCMC) steps, sampling parameters, and trees every 10,000th step. We checked convergence of MCMC chains using Tracer version 1.7.1 (https://beast.community/tracer). We summarized maximum clade credibility trees using TreeAnnotator (https://beast.community/treeannotator) after discarding 10% as burn-in. We submitted the genomes from this study to the Genome Detective for the analysis of the mutational pattern profile using the annotated genome aligner AGA (https://www.genomedetective.com/app/aga). We plotted results in R Studio version 4.2.1 (https://posit.co) using the Lollipop plot.

### ZIKV Neutralization Assays

We measured ZIKV neutralizing antibody titers by a high-throughput ZIKV 50% microneutralization assay (MN_50_), using a wild-type live virus as described previously (*22*). We defined seropositivity as a titer >1:10.

## Results

### Cohort Definition and Sample Assessment for NGS

During June 2017–June 2019, our study recruited a total of 255 persons with ZIKV-confirmed infection in Manaus, Brazil. Among the participants, 99% were enrolled within 1 week after the onset of illness. For this study, genomic analysis included 135 persons with confirmed ZIKV infection experiencing rash, itching, fever, and arthralgia; mean age was 38.27 (+12.97) years (Table 2). Of those 135 persons, 56 participants had >1 sample available, defined as a different specimen at the same visit (5/135) or any specimen at a different study visit (51/135). We sequenced those samples, which yielded a total of 238 ZIKV genomes with a median genome coverage breadth of 90%. Most of these genomes were obtained from plasma and urine samples; a minor proportion (n = 20) were obtained from semen specimens.

**Table 2:** Characteristics of participants included in study of genomic analysis of Zika virus reinfection, Brazil

Characteristics at enrollment	No. (%) participants, n = 135
Age group, y	
18–30	45 (33.2)
31–60	84 (62.2)
>60	6 (4.4)
Sex	
M	45 (33.3)
F	90 (66.7)
Days after symptom onset*	
0–2	44 (32.6)
3–5	68 (50.4)
6–8	22 (16.3)
9–11	1 (0.7)
Clinical signs*	
Macular or papular rash	133 (98.5)
Itching	126 (93.3)
Fever	116 (85.9)
Arthralgia	108 (80)
Nonpurulent conjunctivitis	100 (74.1)
Periarticular edema	92 (68.1)

*Reported at screening visit, which did not require enrollment.

### Phylogenetic Characterization

**Figure 1:**
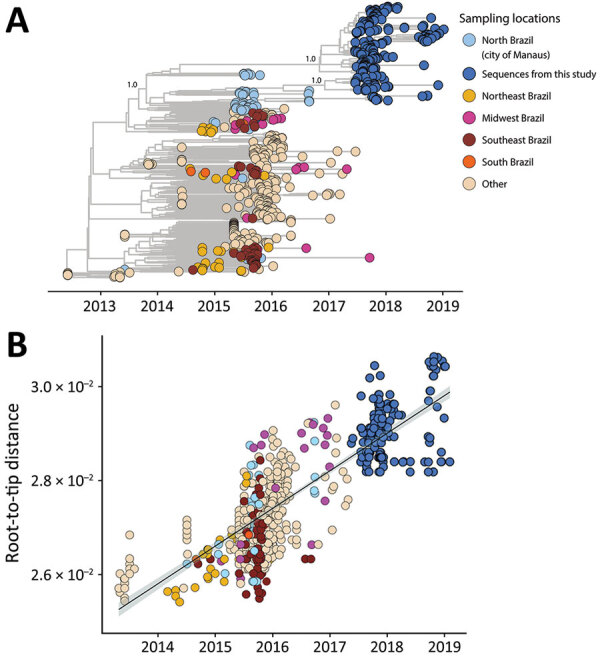
Genomic epidemiology of Zika virus strains obtained from study participants in northern Brazil and reference sequences. A) Time-scaled maximum clade credibility tree of Zika virus Asian lineage in Brazil, including the 238 new genomes generated in this study (dark blue) plus 481 reference strains sampled worldwide. Tips are colored according to the sample source location. Values at nodes represent posterior probability support of the tree nodes inferred under Bayesian evolutionary analysis using a relaxed molecular clock approach. B) Root-to-tip regression of sequence sampling date against genetic divergence from the root of the outbreak clade.

**Figure 2:**
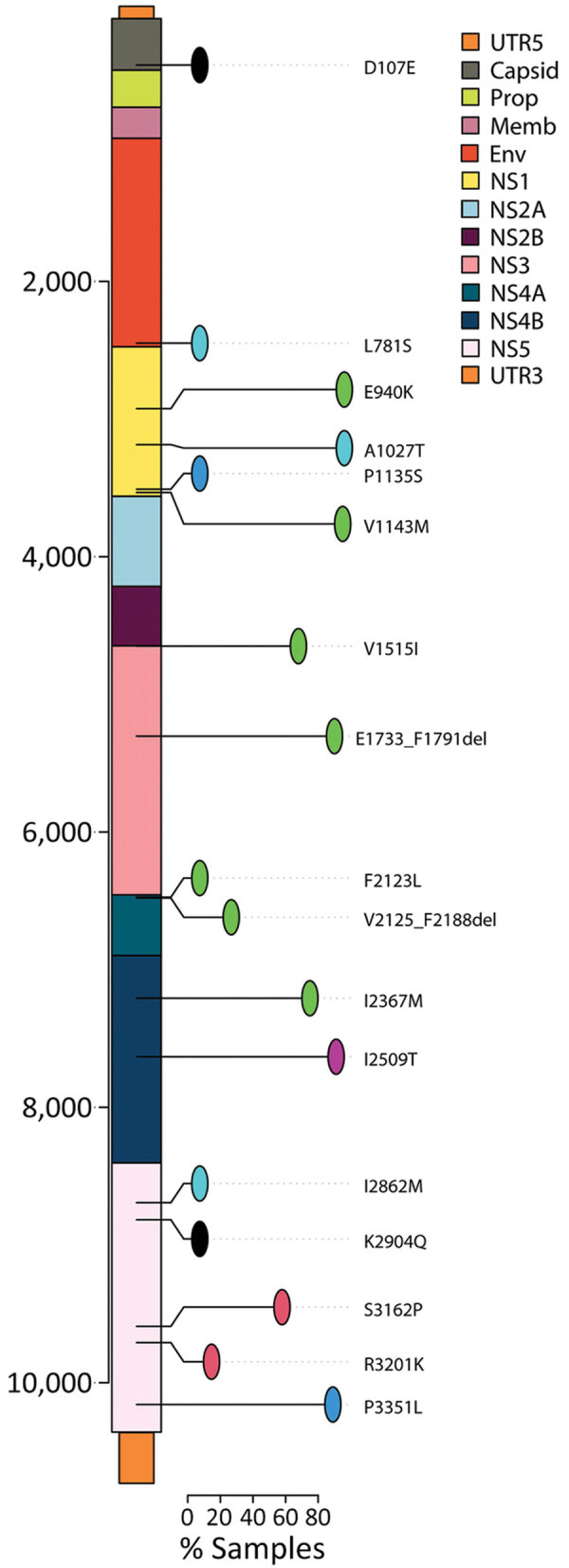
Single-nucleotide variants per gene for Zika virus strains obtained from study participants in northern Brazil. Amino acid changes in the polyprotein are allocated along the genome. Only mutations that appear in >10% (lines) of sequences are shown. Env, envelope; Prop, propeptide; Memb, membrane; NS, nonstructural; UTR, untranslated region.

Initially, our objective was to thoroughly characterize ZIKV circulating from northern Brazil. We observed that all ZIKV strains circulating in Manaus since the beginning of the outbreak, including our newly generated genomes, grouped together in a unique clade within the ZIKV Asian lineage (Appendix 1 Figure 1). In addition, our maximum clade credibility (MCC) tree clustered our generated ZIKV genomes together with viral strains previously isolated in northern Brazil (Figure 1, panel A). From this analysis, we estimated the time of the most recent common ancestor (tMRCA) occurred in late March 2014 (95% highest posterior density range January–August 2014) (Figure 1, panel B). We also explored the collective mutational pattern found in the consensus genomes obtained in this study. Most of the mutations were observed in nonstructural protein (NS) 1 protein (5 in total) and NS5, which also has 5 mutations, although with a lower frequency than NS1 (Figure 2).

**Figure 3:**
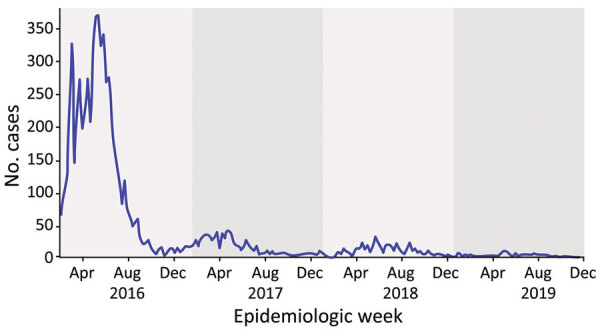
Notified Zika virus cases per week in Manaus municipality, Brazil, 2016–2019, from the Brazilian Ministry of Health. Shading reflects each epidemic year. Data source: https://datasus.saude.gov.br/informacoes-de-saude-tabnet.

Next, we searched for the total number of ZIKV cases reported in Manaus from DATASUS (https://datasus.saude.gov.br/informacoes-de-saude-tabnet), the national health information system that compiles clinical and laboratory-confirmed cases across all the states of Brazil. Our analysis revealed that the initial occurrence of ZIKV cases in Manaus dates to 2016, a significant surge of 6,033 cases that marked the peak of the ZIKV epidemic in northern Brazil. However, after the initial surge in 2016, subsequent waves experienced a significant decrease in the overall number of reported ZIKV cases. That downward trend persisted and reached a notable low point in 2019, when only 126 cases were documented (Figure 3). We concluded that the ZIKV circulating strains in northern Brazil exhibited stability over time, undergoing minimal mutations, contributing to the decline of the epidemic.

### ZIKV Within-Host Genetic Diversity

**Figure 4:**
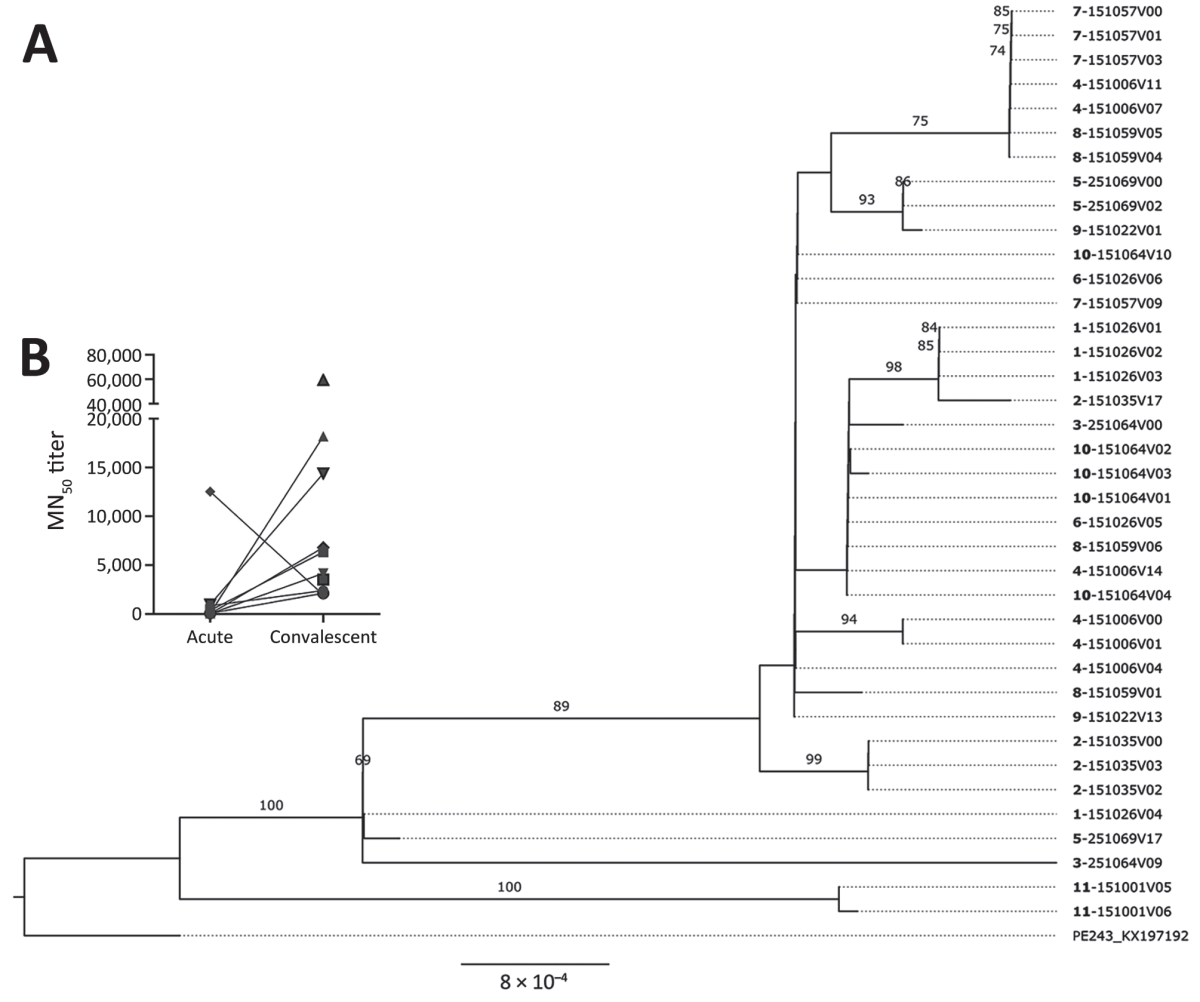
Phylogenetic analysis of study participants persistently infected with Zika virus, Brazil. A) Maximum-likelihood phylogenetic tree of persistent samples. The phylogenetic tree shows all 10 participants with confirmed persistent infection. Boldface indicates participant identification numbers; visit numbers (V) are indicated. Multiple identification numbers represent multiple genomes obtained from the same participant at different time points. Scale bar indicates number of nucleotide substitutions per site. Numbers on the branches indicate Shimodaira–Hasegawa approximate likelihood ratio test after 1,000 replicates. B) Neutralizing antibody titers from acute and convalescent samples, as analyzed from persistently infected participants. MN_50_, 50% microneutralization.

We followed our study protocol, specifically designed to investigate the persistence of ZIKV in body fluids, to assess virus diversity among persons who remained persistently infected. Although there is no consensus in the literature, we defined ZIKV persistence as any participant with ZIKV-positive rRT-PCR detection within 30 days after its initial ZIKV confirmation. By applying this criterion, we identified 10 patients who had >1 positive persistent sample from plasma, urine, or semen. Individual temporal phylogenetic analysis grouped those ZIKV-persistent genomes into 2 major clades. For 5 of the patients, all their samples grouped into a single clade in the tree (Figure 4, panel A); those clusters indicated limited viral diversity and maintenance of a single viral lineage through time in these persistently infected persons, independent of the type of specimen analyzed. Because ZIKV neutralizing antibodies (ZIKV-NAb) are highly protective and increasing titers from acute to convalescent phase are usually linked to viral clearance, we then assessed the levels of ZIKV-NAb. Our results showed that almost all the 10 persistently ZIKV-infected participants responded with higher levels (>2,000) of ZIKV-NAb by 30 days after disease onset (Figure 4, panel B), indicating a strong neutralizing antibody response at the convalescent phase. Those results eliminated the possibility of a dysregulated immune response as a cause of persistent ZIKV infections.

**Figure 5:**
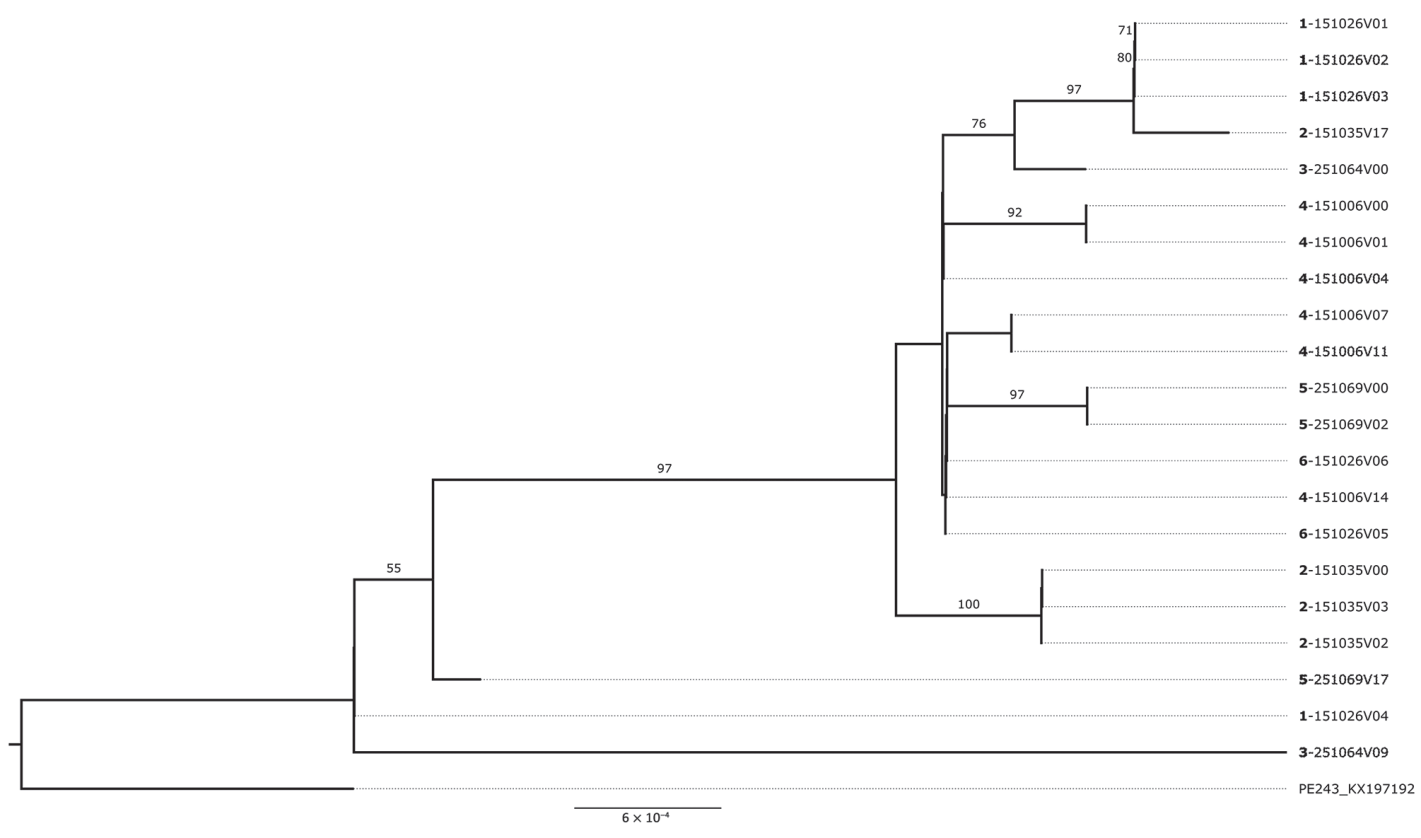
Maximum-likelihood phylogenetic tree supporting Zika virus reinfection among study participants in northern Brazil. The tree shows the 5 participants with divergent samples in which coinfection by different ZIKV genomes was inferred by phylogenetic reconstruction. Divergent samples from the same participant were grouped separately in the tree. Boldface indicates participant identification numbers; visit numbers (V) are indicated. Scale bar indicates number of nucleotide substitutions per site. Numbers on the branches indicate Shimodaira–Hasegawa approximate likelihood ratio test after 1,000 replicates.

**Figure 6:**
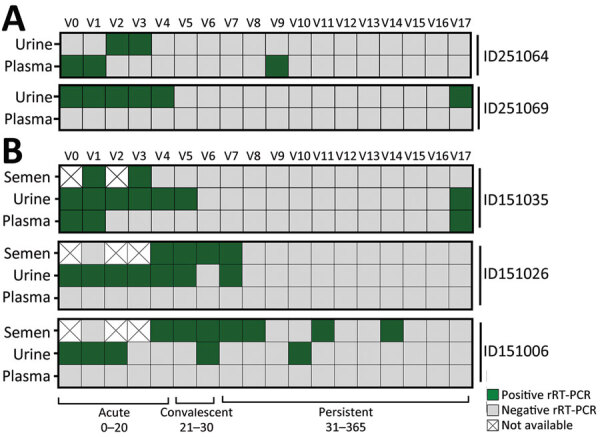
Zika virus rRT-PCR results from plasma, urine, and semen (when applicable) specimens supporting reinfection among female (A) and male (B) study participants in northern Brazil. Each square represents an analyzed specimen according to the schedule from study visits (Table 2). ID, participant identification; r-RT-PCR, real-time reverse transcription PCR; V, visit number.

Our phylogenetic analysis also showed 5 participants with ZIKV genomes clustering in distinct clades or subclades in the tree (Figure 4, panel A; Figure 5; Appendix 1 Figure 2), which suggests the presence of divergent viral genomes within the same participant over time. Those participants had highly supported minor variants (approximate likelihood ratio test >70%) that were not consistently found among all samples from the same person and showed no consistent pattern of minor variant sites accumulation over time (Appendix 2). We hypothesize that the presence of such temporally divergent ZIKV genomes in the same person suggests a reinfection event by a distinct ZIKV clade. Thus, to further assess whether those participants were reinfected, we checked their rRT-PCR results. We observed that 1 participant (ID251064) had a continuous rRT-PCR–positive result up to 8 days after the initial ZIKV infection; viral RNA was not detected until study visit 8 (61–90 days after disease onset), when a ZIKV rRT-PCR result was again positive in plasma (Figure 6). Two participants (ID251069 and ID151035) tested positive for ZIKV RNA in either plasma or urine for up to 21 days after ZIKV confirmation. Those participants then remained ZIKV-negative for 10 months but returned to positivity at the last study visit, performed 311–360 days after disease onset (Figure 6). The rRT-PCR–positive samples indicating reinfection exhibited the highest degree of divergence in terms of the ZIKV genome compared with the acute phase–sequenced samples obtained from the same participants (Figure 5).

**Figure 7:**
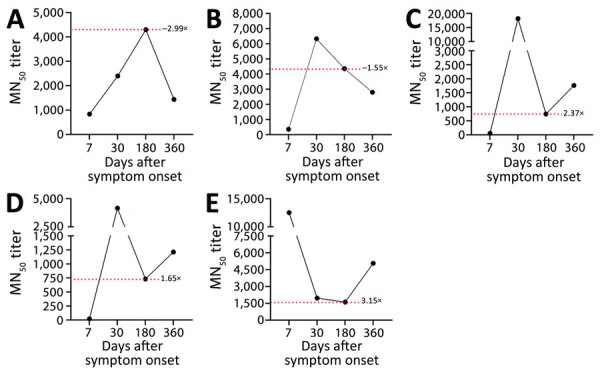
Zika virus neutralizing antibody titers from acute and convalescent serum samples supporting reinfection among 5 study participants in northern Brazil. A) Participant ID151006; B) participant ID151026; C) participant ID151035; D) participant ID251064; E) participant ID251069. Dotted lines and red numbers represent fold changes in titers as calculated from the 180-day and 360-day intervals. MN_50_, 50% percent microneutralization assay.

Because reports on the genomic characteristics of ZIKV isolates from 2017 onward in northern Brazil are lacking, we conducted a complementary analysis of our own dataset that revealed the presence of these exact genomes associated with reinfection within the population. Of note, we observed the presence of these same genomes in multiple samples from our cohort (Appendix 1 Figure 2), providing strong evidence that the viruses were circulating both temporally and geographically. Finally, to support the assumption of reinfection, we analyzed the levels of ZIKV-NAb at 7, 30, 180, and 360 days after disease onset, assuming that antibody titers would vary among initial infection and reinfection, mirroring rRT-PCR results. We observed that all but 1 participant (ID251069) responded with increased levels of ZIKV-NAb at the convalescent phase of the disease (30 days after symptom onset) (Figure 7). At 180 days after onset we observed a decay in ZIKV-NAb levels at an interval when the primary infection was already cleared. All 3 potentially reinfected participants (ID251069, ID151035, ID251064) responded with a second increase in the levels of ZIKV-NAb at the last interval analyzed (Figure 7), which was preceded by viral RNA redetection in plasma or urine specimens. We also discarded other arbovirus infections as an inducer of ZIKV-NAb response because our study protocol was based on a validated multiplex rRT-PCR and none of the participants tested positive for either dengue or chikungunya virus. Other well-known circulating arboviruses in northern Brazil are Oropouche virus (OROV) (*23*) and yellow fever virus (YFV) (*24*). To date, no documented reports have indicated that OROV is capable of eliciting a ZIKV-specific antibody response. Furthermore, most study participants had prior YFV vaccination, so it is unlikely that they had become infected; thus, we have effectively ruled out YFV as a potential confounding factor in relation to the antibody response associated with ZIKV reinfection. Of note, none of the 3 potential reinfection cases reported symptoms typically associated with ZIKV infection, as confirmed through a comprehensive anamnesis conducted during each study visit at our study clinic. Collectively, our data strongly support the occurrence of reinfection events in at least 3 healthy persons residing in a ZIKV-endemic area in Brazil.

## Discussion

Given the number of ZIKV cases registered at the peak of the 2016 epidemic in the northern and other regions of Brazil (*5*), added to the risk for new outbreaks, it is critical to study ZIKV evolution and its potential for adaptation to vertebrate hosts. Moreover, virus persistence may exert high evolutive pressures that contribute to virus evolution and transmission. In our study, we showed that the obtained ZIKV genomes clustered together with other ZIKV Asian strains previously isolated from northern Brazil, suggesting that this strain persisted locally through natural transmission and was kept circulating among humans until August 2019 or later. We also found that the temporal circulation of ZIKV in Manaus started a descending curve, supported by a decreasing number of cases registered after the peak of the epidemic in 2016. Thus, based on a seroprevalence study from northeastern Brazil showing that the ZIKV antibody prevalence reached a peak of 63% from 2015 to 2016 (*25*), in addition to other studies showing a high ( >60%) seroprevalence of ZIKV antibodies in the general population (*26*–*28*), we hypothesize that, within a single year, community immunity was enough to constrain virus circulation. In fact, our findings are consistent with a lower reproduction number (R0) since late 2016 in Salvador (*25*), corroborating mathematical modeling studies showing that ZIKV epidemics would be over in 3 years from its introduction in 2016 (*29*).

Long-term cohort studies can provide longitudinal data on individual virus diversity, virus evolution, clinical symptoms, and immunological outcomes, and so are crucial to better understanding ZIKV natural history. We found evidence of limited virus diversity over time from persistently ZIKV-infected persons, a feature that has also been observed by other independent studies (*18*,*19*). Thus, we can suggest that the evolutionary rates and selection pressures acting on ZIKV are moderate, affecting virus evolution and adaptation to local populations. In fact, similar to a previous report (*30*), we estimated the ZIKV whole-genome evolutionary rate at around 1.18 × 10^−3^ substitutions/site/year. Arboviruses primarily spread through horizontal transmission between arthropod vectors and vertebrate hosts. As a result, virus evolution is restricted by the need for optimal replication in one host, which may compromise their adaptation in the other (*31*), contributing to the short-term and long-term reduced number of adaptative mutations observed. In addition, various factors, including the short duration and low viremia observed in naturally infected persons (*32*), contribute to limiting ZIKV diversity. Consequently, our findings indicate that ZIKV displayed a relatively stable genome evolution over time and did not undergo rapid changes or diversification during the epidemic in northern Brazil.

The most notable finding of our study is the identification of reinfection events, which is highly intriguing. Given that the ZIKV epidemic in Brazil originated from a single virus strain, and combined with the observation that the virus has remained relatively stable over time, tracking reinfections becomes a challenging task. Complicating matters further, most infections are asymptomatic or cause only mild symptoms, such as fever, rash, and itching (*33*). As a result, persons who have been potentially reinfected may have gone unnoticed, especially considering that mild symptoms often do not prompt persons to seek medical attention. Although reinfections are extremely difficult to confirm when there are only very similar phylogenetically strains causing an outbreak, we detected divergent viruses in ZIKV-infected persons who provided longitudinal samples, which suggests a subsequent and distinct infection event.

Monitoring community virus circulation plays a crucial role in confirming infections within a population. As extensively explored for several other viruses (*34*), mapping diversity in a community can provide valuable information for confirming infection cases and understanding the dynamics of an outbreak. Thus, by sequencing viral genomes, it is possible to identify an specific strain or variant of the virus present in an individual or a community (*35*). Despite detecting the presence of these same reinfection-associated ZIKV genomes in other participants of our cohort, temporally and geographically confirming the circulation of these genomes in that population, there is a notable absence of independent studies validating the presence of these genomes at the time we detected potential reinfection cases. Most of the investigations from other groups were conducted during the early stages of the ZIKV outbreak; therefore, the literature lacks reports that describe the characteristics of the viruses circulating from late 2017 onward.

Confirmation of reinfection events based solely on molecular detection may introduce uncertainties because of the possibility of cross-contamination during sample processing. To address this concern, we conducted an assessment of ZIKV antibody response. By measuring the levels of ZIKV-NAb over time, we can add a deeper understanding of ZIKV infection dynamics, immune response effectiveness, and the potential for future reinfections (*36*). Here, we observed that 3 persons responded with a second increase in ZIKV-NAb levels, which was temporarily associated with rRT-PCR positivity at a late time point after the initial infection. We discarded other arbovirus infections as a cause of secondary ZIKV-NAb increase because all the participants from our cohort were tested in a multiplex rRT-PCR and none of them were positive for dengue or chikungunya virus. We also discarded YFV infection and YFV vaccination because our study participants were previously vaccinated against YFV. We also assumed that these reinfection events were very mild, mostly manifesting as an asymptomatic disease, because no symptoms were reported.

Our findings hold significant implications for public health, epidemiology, clinical practice, and diagnostics. However, the frequency of reinfections during the latest ZIKV outbreaks remains uncertain. Our study emphasizes the critical role of ongoing genomic surveillance in viral infections to enhance public health interventions. Therefore, we underscore the necessity of implementing continuous surveillance strategies, which are vital for monitoring the evolutionary changes of viruses over time and gaining a comprehensive understanding of arbovirus diversity.

Appendix 1 Additional information about genomic analysis indicating Zika virus reinfection, Brazil.

Appendix 2 Data for 5 participants in study of Zika virus reinfection, Brazil.
