# Supplementary material for: Evidence of Zika Virus Reinfection by Genome Diversity and Antibody Response Analysis, Brazil
Source: Emerg Infect Dis. 2024 Feb;30(2):310–20. doi: 10.3201/eid3002.230122 (PMC10826783; doi:10.3201/eid3002.230122)
Supplement: Appendix 1 — Additional information about genomic analysis indicating Zika virus reinfection, Brazil. [file 23-0122-Techapp-s1.pdf]

Article DOI: http://doi.org/10.3201/eid3002.230122

EID cannot ensure accessibility for supplementary materials supplied by authors. Readers who have difficulty accessing supplementary content should contact the authors for assistance.

Evidence of Zika Virus Reinfection by Genome Diversity and Antibody Response Analysis

Appendix 1

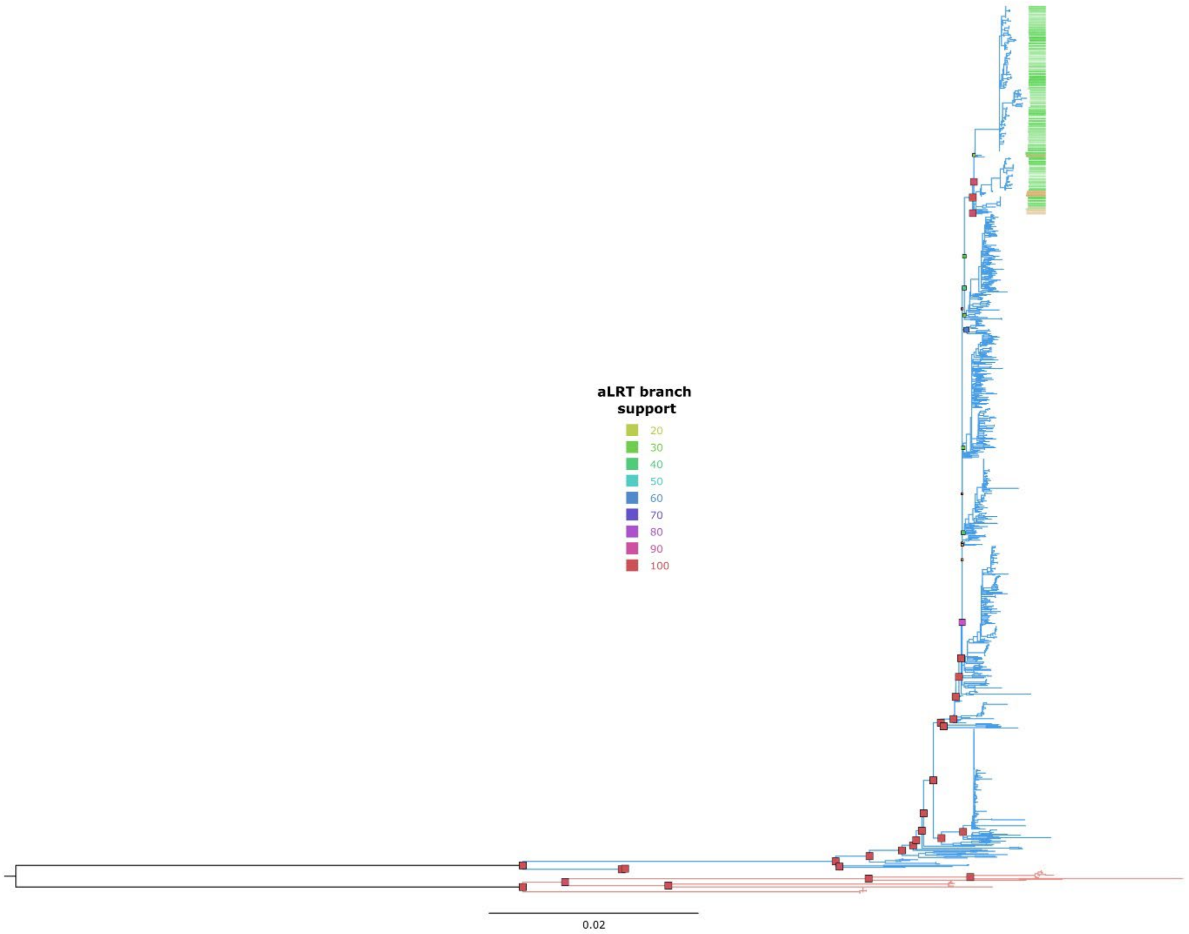

Appendix Figure 1. Maximum likelihood phylogenetic tree reconstructed based on the coding nucleotide region of eight hundred fourteen ZIKV genomes sequences downloaded from ViPR database and all genomes generated in this study. Red and blue bars denote genomes belonging to African and Asian ZIKV lineages while green bars are samples generated in this study.

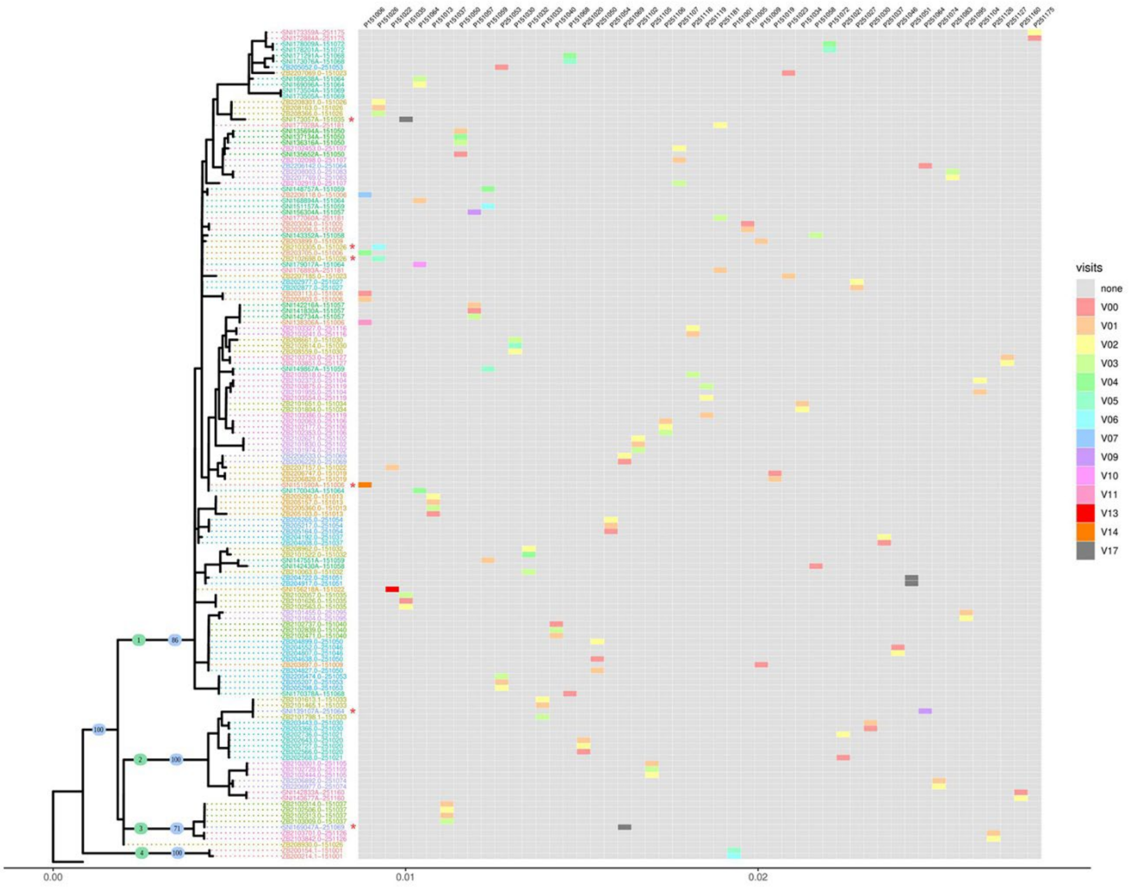

Appendix Figure 2. Maximum likelihood phylogenetic tree including all ZIKV genomes generated in this study. Participant identifications (IDs) are color-coded in the tip labels, the same color represents multiple samples obtained at different time points from the same participant. Green numbers in the tree branches (1 to 4) represent the different ZIKV clades as identified in our analysis and blue numbers are the aLRT branch support of each defined clade. Columns represent the study participants with at least one persistent associated sample. Color-coded columns represent the study visit, as described in Table 1. Reinfection-associated genomes are marked with a red asterisk. Pattern of minor variant accumulation.
